# Patient’s satisfaction in physiotherapy outpatient departments of Amhara regional comprehensive specialized hospitals, Ethiopia

**DOI:** 10.1186/s12913-022-08338-y

**Published:** 2022-08-08

**Authors:** Yisak Girma Guadie, Alemu Kassaw Kibret, Kedir Sany Adem, Ermias Solomon Yalew

**Affiliations:** Department of Physiotherapy, School of Medicine, College of Medicine and Health Sciences, University of Gondar Comprehensive Specialized Hospital, University of Gondar, P.O. Box: 196, Gondar, Ethiopia

**Keywords:** Satisfaction, Physiotherapy, Outpatient service, Ethiopia

## Abstract

**Background:**

Satisfaction is basically the state of being gratified and act of fulfilling one's need or desires. Nowadays, evidence-based practice concept is prevailing and there has been a growing interest in the measurement of patient satisfaction in healthcare research. Patient satisfaction surveys provide several benefits for healthcare professionals. Assessing patient’s satisfaction with physiotherapy service could generate knowledge that can utilized for improving or maintaining quality service. Although a wide coverage and high emphasis givens to patient satisfaction studies in developed counties, there are few research’s done in Africa. This study aimed to assess satisfaction in physiotherapy service and identify predictors that affect satisfaction among patient treated in physiotherapy outpatient department.

**Objectives:**

To assess satisfaction in physiotherapy service and identify associated factors among patients in physiotherapy outpatient department in Amhara regional state comprehensive specialized hospitals.

**Method:**

Institution based cross-sectional study was conducted among physiotherapy outpatients from April to June 2021, at three selected Comprehensive specialized hospitals. Data were collected by interviewing participants using semi-structured questionnaire. Patient satisfaction was determined by using Medrisk tool. Data were analyzed by using descriptive statistics and bivariate and multivariable logistic regression method were used to identify predictor factors.

**Result:**

A total of 409 participants with a response rate of 95% were included in this study. The overall satisfaction among physiotherapy outpatient attendee was 50.1% with 95% CI (46.2–55.7). Pain level (AOR = 5.59 95%CI (2.58–12.1), longitudinal continuity of care (AOR = 3.02 95%CI (1.46–6.62) and self-rated health improvement (AOR = 3.76, 95% CI (1.78–7.94) were significantly associated factors.

**Conclusion:**

The overall satisfaction in this study were found to be low in Amhara regional state comprehensive specialized hospitals. pain level, self-rated health improvement and longitudinal continuity of care were significantly associated factors.

## Background

Patient satisfaction with physiotherapy service is a relatively under-utilized outcome measure in the Physiotherapy health care system. Asking for patients’ feedback in a systematic way and utilizing those feedback as well to make some appropriate changes accordingly in the delivery of care is a major problem in the physiotherapy care setting [1, 2]. Most of the satisfaction studies in this field concentrate on patient outcomes and there is a scarcity of physiotherapy service satisfaction studies. Although there are some efforts and some studies regarding client satisfaction studies in physiotherapy services study indeed, it is concentrated in a developed country and well-established centers, and little is known about services in the developing countries [3]. This in turn prevents us from knowing key factors like; whether patients are satisfied with the service or not, what patients value most from the service, which needs adjustment etc. eventually fail to fill these gaps, and identifying the contributing factors leads to patient dissatisfaction, lack of adherence and, most importantly patients loos the benefit from the service [3–5].

Satisfaction is basically the state of being gratified and an act of fulfilling one's needs or desires. Satisfaction of patients accurately shows the patient's attitude about the health care services and how patients perceive health services or health care providers and patient satisfaction governs the value and importance of health care [5].

Nowadays, the patient-centered care approach is the recent evidence-based practice among leading standard healthcare systems and Patient satisfaction is commonly regarded as the most important component of the concept [6]. There has been a growing interest in the measurement of patient satisfaction in healthcare research and this is a pragmatic move towards patient-centered care. Internationally, many governments, healthcare systems or institutions, and patient-led advocacy organizations have established patient satisfaction as a proxy measure of care appropriateness, efficacy, quality, and feasibility [7].

Patient satisfaction within the rehabilitation is needed as it affects the success of therapeutic treatment and adherence to treatment management. Satisfaction with healthcare services often impacts the quality of care and has a direct impact on the marketability and competence of essential services. Although patient satisfaction is multidimensional and can be judged by emotion, rehabilitation professionals can use the feedback to improve or adjust practices [8, 9].

Physiotherapy is a drug-free therapeutic system of medicine that comprises examination, treatment, advice, and instructions in connection with movement dysfunction, bodily malfunction, physical disorder, disability, healing and pain from trauma and disease, and physical and mental conditions using physical agents [10, 11]. Physiotherapy as a profession has several unique characteristics that may influence patient satisfaction in the treatment process which include the contact hours with the patient often takes longer than a routine medical visit, it involves more physical contact, and the therapy usually requires the patient's active participation and the treatment by itself may cause temporary pain that may be perceived as physically threatening. Thus, assessing patient satisfaction in physiotherapy needs to consider these components [12].

Assessing patient satisfaction and taking measures in components in which patients were dissatisfied has much importance in Physiotherapy service. This is evident by the fact that satisfied patients are more likely to adhere to treatment and achieve a better quality of life. Further, this fact is justified by the physiotherapist establishing a relationship with the patient revealing interest in his improvement, clarifying his doubts, and explaining what was done in the treatment. Thus, a therapist-patient relationship of trust and safety is established, motivating the patient during treatment to achieve improvement in their pain. On the other hand, dissatisfied patients may discontinue the treatment and influence other clients by creating a negative image of the center. Finally, this leads to potential economic harm to both the patient and the health center. because dissatisfied patients spread negative views and for better services [6, 13].

There are different factors that contribute to patient satisfaction in physiotherapy services including sociodemographic factors ( age, sex, level of education, residence, and Level of income), clinical factors (onset of injury, adequate duration, and frequency of treatment, outcome, and continuity of care) and service-related factors(cost, follow-ups, and privacy) [14, 15]. Thus, by considering these factors assessing Patients’ views about their health care is considered one of the three key elements of evidence-based physiotherapy practice [16, 17].

Despite few pieces of research done in Africa, to extent of our knowledge, there is a paucity of research in Africa including Ethiopia. Therefore, the purpose of this study was to assess patient satisfaction and associated factors of physiotherapy service among physiotherapy outpatient attendees in Amhara regional comprehensive specialized hospitals.

## Method and materials

### Study design, period, and setting

An Institutional based cross-sectional study was conducted from April to June 2021 at the University of Gondar Comprehensive Specialized Hospital (UOGCSH), Felege Hiwot Comprehensive Specialized Hospital (FHCSH), and Dessie Comprehensive Specialized Hospital hospitals (DCSH). The above three hospitals were selected purposefully for their large catchment area and similarity to physiotherapy outpatient service standards. University of Gondar comprehensive specialized teaching hospitals physiotherapy outpatient clinic was established in 2002 G.C by Netherlands collaboration and is the first physiotherapy teaching center in Ethiopia. The department of physiotherapy has seen 150 patients per month. And contributing by physiotherapy treatment and rehabilitation programs in Gondar and surrounding catchment areas. Felege Hiwot comprehensive specialized hospital was established in 1963 as a district hospital and upgraded to a referral hospital in 1994 in a physiotherapy outpatient clinic around 100 patients get the service within a month. Dessie Comprehensive Specialized hospital is one of the oldest hospitals in the Amhara region, located in Dessie which is 401 KM far from the northeast of Addis Ababa, the capital city of Ethiopia. The hospital is serving approximately seven million people in the catchment area. the catchment population is about 7 million.

### Source population, study population, inclusion and exclusion criteria

Patients aged 18 and above and who had a minimum of 4 treatment sessions at the physiotherapy outpatient department were the study’s source population [18]. And all adult patients who were treated in outpatient physiotherapy at Gondar, Bahir Dar, and Dessie comprehensive specialized hospitals during the data collection period were the study population. All adult patients who had at least 4 treatment sessions in the outpatient physiotherapy department were included in this study. Whereas Patients who were on physiotherapy treatment for less than 4 visits and individuals with medically known or recognized psychological issues and individuals with commination deficiency were excluded from the study.

### Sample size determination and sampling procedure

Since there is no similar study done in Ethiopia, sample size was calculated by using single population proportion formula; 95% confidence interval, 5% precision, 10% non-response, and contingencies. The sample size was estimated as follows:

$$\mathrm n=\left(\frac{\mathrm{za}}2\right)^2\mathrm p\left(1-\mathrm p\right)/\mathrm d2$$

$$\mathrm{n}=\left(1.96\right)2*\left(0.5\right)*\left(0.5\right)/{\left(0.05\right)}^{2}=384.16$$

*n* = 384.2. By adding 10% non-respondent rate and contingency the final sample size is 424.

**Fig. 1:**
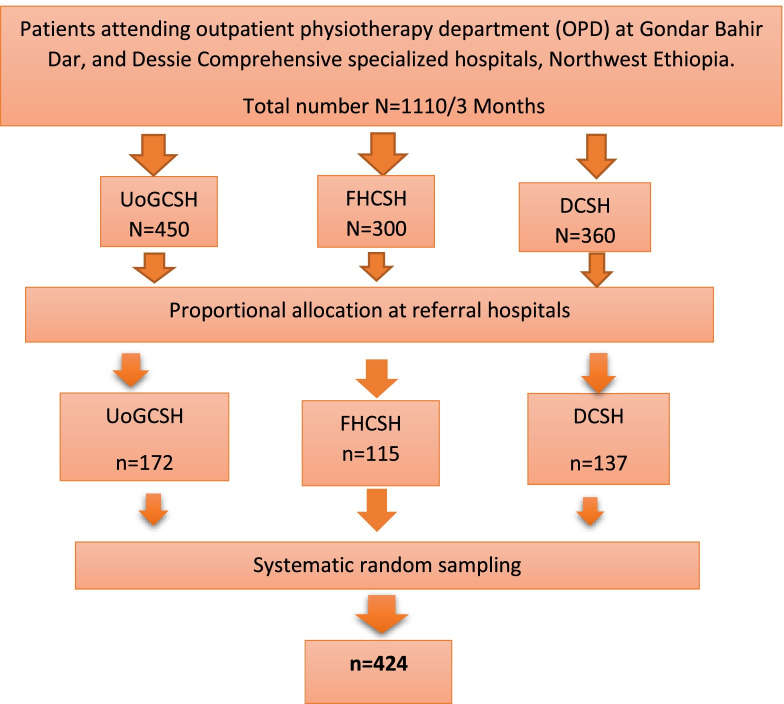
Schematic representation of sampling procedure for patient satisfaction at Comprehensive specialized hospitals, North Ethiopia, 2021

Systematic random sampling was applied to select the study subjects. Study participants were selected from the three selected comprehensive specialized hospitals in the Amhara region, North Ethiopia. Proportional allocation was done from each of the selected hospitals to ensure the representativeness of study participants. 172, 115, and 137 participants were selected from UOGCSH, FHCSH, and DCSH respectively. The “K” value was calculated to be 2.62 and rounded to 2, (*N* = a total number of patients averagely expected from the three hospitals within three months 1110, *n* = 424; K = 1110/424 = 2.62). To draw the 1st sample from the first two participants, lottery methods were used, and then the rest samples were chosen in every Kth interval for each hospital of outpatient physiotherapy department. (Fig. 1 shows the sampling procedure).

### Operational definitions

This study used the tool of MedRisk (a 20-item version) to measure outpatient physiotherapy services. The MedRisk is the most accepted and widely used tool with good validity and reliability, Cronbach’s alpha 0.83. MedRisk tool is used to collect data related to patient satisfaction with physiotherapy services. It is comprised of 20 items with a rating scale of 1 to 5: Not satisfied at all (1); unsatisfied (2); More or less satisfied (3); satisfied (4); Very satisfied (5) [19]. Generally, the items are divided into 5 components: communication and respect, convenience, quality time, person-focused care, and overall(global) satisfaction component [20]. The tool was adopted without significant changes and a pretest was done to check if there was a transcultural barrier and we did not find difficulty in understanding all questions. Medians were used as a cut point to determine patients as satisfied or not satisfied since the data was not normally distributed. Thus, who score above the median are considered satisfied and those who score below the median are regarded as unsatisfied.

*Longitudinal continuity of care* is defined as the practice in which a patient is seen by the same practitioner for the entire course of treatment [21].

Pain level: pain level was measured by using the Visual Analogue Scale which is a reliable tool to assess both acute and chronic conditions. The score ranges from 0 up to 10, 0 = no pain, 1–3 = mild pain, 4–6 = moderate pain, 7–10 = severe pain [22].

### Data collection instrument

An interviewer-administered questionnaire was used to assess patients' background information, which included socio-demographic questions. Questions were adapted from previously published articles with similar studies and methodology. The questionnaire comprises three components the information on socio-demographic and clinical characteristics related questionnaire and, satisfaction related questions. The questionnaire was first written in English by language experts and authors at the University of Gondar, who double-checked the questions for consistency. Language experts translated the English version of the questionnaire into the local language (Amharic) and then back into English. The differences between the original and translated questionnaires were compared, and the discrepancies were assessed and corrected as needed.

Face-to-face interviews and a review of patient records were used to collect the data. Three senior physiotherapists were in charge of data collection. The principal investigator gave the data collectors a two-day intensive training on how to approach study participants, how to use the questionnaire and guidelines, and data collection methodologies. The investigators kept a careful eye on the data collection procedure and checked the obtained questionnaire on a daily basis for accuracy, completeness, and consistency.

### Data analysis procedure

Epi-data version 4.6.0.4 was used to code and enter the data, which was then exported to SPSS for Windows version 20. Data cleaning and missing values were checked. Descriptive statistics were used to explain demographic data. Binary logistic regression was used to examine the relationships between dependent and independent variables. To control the effects of potential confounders, independent variables with *p*-values < 0.2 in univariable analysis were exported to multivariable logistic regression analysis.

The significance of associations was computed with a *P*-value < 0.05, with an adjusted odds ratio (AOR) of 95% confidence interval (CI). To check the model fitting Hosmer–Lemeshow goodness of fit test was checked which was a *P*-value of > 0.05.

## Result

### Socio-demographic characteristics of participants

A total of 409 patients participated in the study making the response rate 95%. The majority of participants were from Gondar followed by Dessie and Bahir Dar (166,132 and 111 participants respectively). Most of the participants 318(77.8%) were from urban. The median age of participants was 34 years with an interquartile range (IQR) of 24 to 47 years. Of the total participants, orthodox Christians in religion were 295(71.1%), males in their sex 257(62.8%), and married in their marital status 238(58.2%). Less than half of the participants 171(41.8) had diplomas and above. Of the total participants, 158 (38.6%) were civil servants while 134 (32.8%) work in private.

### Clinical charactherstics of study participants

The mean (SD) times, that participants spent in the hospital while they were treated were 33 min (SD = 12.6). In contrast, the shortest time was 10 min and 60 min was the longest time. Table 1 shows the clinical characteristics of study participants.

**Table 1:** Clinical characteristics of study participants at UOGCSP, FHCSH and DCSH specialized, North Ethiopia, 2021 (*n* = 409)

Variables	Frequency	Percent
Duration of the problem		
0–12 weeks	139	34.0%
13- 24 weeks	80	19.6%
> 24 weeks	190	46.4%
Compliant of pain		
Yes	314	76.8%
No	95	23.2%
**Pain severity**		
Mild	81	25.8%
Moderate	109	34.7%
Sever	124	39.5%
**Site of injury**		
Face and neck	35	8.6%
Back	89	21.8%
Upper limb	87	21.3%
Lower limb	107	26.2%
Multiple area	91	22.2%
**Number of visits**		
4–5 times	107	26.2%
6–8 times	138	33.7%
9–1 1times	51	12.5%
> 11 times	113	27.6%
**Treatment time(minutes)**		
0–15	33	8.1%
16–30	186	45.5%
31–60	189	46.4%
**Post treatment health status**		
Better	260	63.6%
No change or Worse	149	36.4%
**Previous physiotherapy visit**		
Yes	100	24.4%
No	309	75.6%
**Referral system**		
Self-referred	90	22.0%
Referred by health worker	319	78.0%

### Factors related to service

In terms of service-related factors, the Majority of participants 327(80%) were satisfied with the cost of treatments, and the lowest satisfaction was observed with maintaining privacy during treatment 243(59.4%). With regard to follow-up and appointment, 283(69.2%) were satisfied and 257 (62.8%) of participants were satisfied with the services they received starting from the entrance until they reached physiotherapy OPD.

### Proportion of patient satisfaction with outpatient physiotherapy service

**Fig. 2:**
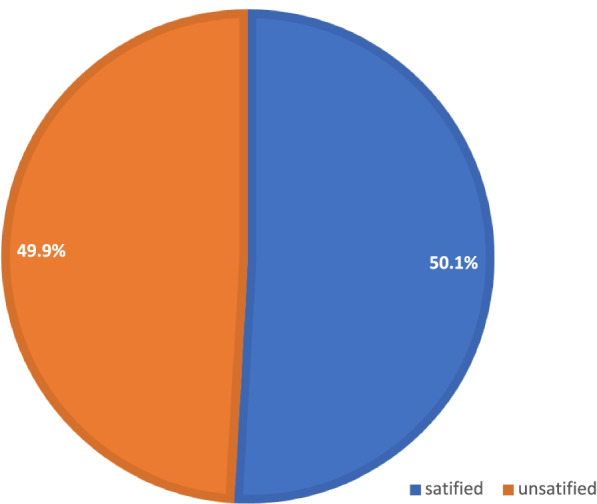
Proportion of patient satisfaction with physiotherapy service among physiotherapy outpatient attendees (*n* = 409) in Amhara regional state comprehensive specialized hospitals, 2021

From the total of participants half of them were satisfied with outpatient physiotherapy services (Fig. 2: shows the proportion of satisfaction with physiotherapy service).

### Factor associated with overall satisfaction related to physiotherapy service

By using bivariate logistic regression analysis 7 factors were found to be significantly associated with the overall satisfaction level. In multivariable logistic regression, pain level, longitudinal continuity of care, and self-rated improvement were significantly associated with overall satisfaction using the forward stepwise (LR) method. The model was checked by the test of by Hosmer Lemeshow test (0.3).

In this study among participants who had pain those who experienced mild pain were 5.59 times more satisfied compared to those who had severe pain (AOR = 0.5.59 CI 2.58–12.1).

On the other hand, participants who were treated by one Physiotherapist were 3.02 more satisfied compared to participants who were treated by Two and more physiotherapists (AOR = 3.02 CI 1.46–6.62). Similarly, based on the self-rated improvement study participants who rated their health condition better after starting treatment were 3.76 times more satisfied compared to those who rated their health status as no change or worse (AOR = 3.76 CI 1.78–7.94) (Table 2).

**Table 2:** Multivariable logistic regression analysis on factors associated with satisfaction among physiotherapy outpatient attendees in Amhara regional state compressive specialized hospitals, 2021 (*n* = 409)

Variable	Satisfied		OR (95 CI)	
	Yes	No	COR (95% CI) *p* value	AOR (95% CI)
**Gender**				
Male	112	145	1	1
Female	96	56	2.20(1.47–3.35)	1.21(0.62–2.36)
**Marital status**				
Single	51	87	1	1
Married	143	95	2.56(1.66–3.95)	1.23(0.59–2.58)
Widowed and Divorced	14	19	1.25(0.58–2.72)	1.48(0.43–5.03)
**Pain severity**				
Mild	55	26	9.80(5.99–18.89)	**5.59(2.58–12.11) **^**a**^
Moderate	78	31	11.66(6.27–21-7)	4.32(0.89–10.37)
Sever	22	102	1	1
**Duration of the problem**				
Weeks	92	47	2.87(1.87–4.52)	0.68(0.32–1.46)
13–24 Weeks	39	41	1.39(0.82–2.36)	0.64(0.28–1.47)
> 24 Weeks	77	113	1	1
**No of PT treated by**				
One	164	66	7.62(4.88–11.88)	**3.02(1.46–6.26) **^**a**^
Two and more	44	135	1	1
**Self-rated improvement**				
Better	187	77	12.15(7.15–20.66)	**3.76(1.78–7.94) **^**a**^
No change or worse	29	116	1	**1**
**Previous PT visit**				
Yes	57	43	1.38(0.88–2.18)	1.43(0.96–2.87)
No	151	158	1	1

1 = reference category *AOR* Adjusted odds ratio, *CI* Confidence interval, *COR* Crudes odds ratio

^a^ = statistically significant at *p* < 0.05

## Discussion

The aim of this study was to find out the proportion of satisfaction in physiotherapy service and identify the associated factors among patients in the physiotherapy outpatient department in the Amhara region comprehensive specialized hospital. The overall magnitude of satisfaction with physiotherapy service was 50.1% (AOR:46.2–55.7). Pain level, longitudinal continuity of care, and self-rated improvement were significantly associated with overall satisfaction.

The proportion of satisfaction in this study was lower compared to studies done in Kenya( 96.5%) [23], and Legos(97.9%) among physiotherapy outpatient attendees [18]. This difference might be due to differences in study participants, in Kenya study participants were recruited from Low back pain patients and the study participants’ cases were exclusively musculoskeletal and did not include participants with neurological cases which in fact is known to decrease satisfaction because of the slow and incomplete recovery nature of some neurological cases. In addition, these discrepancies might be due to differences in the study area, study participants in the Legos study were recruited from teaching hospitals which might create quality differences in service provision compared to ours. The other likely reasons might be due to differences in methodology, in Legos’s study they used different tools as outcome measures (Physical Therapy Patient Satisfaction Question).

Similarly, this study was lower than the study done in Malaysia in 2013(95.2%) [10] in which the majority of the study participants were older in age, and from the previous studies, it is scientifically believed that older patients have high patient satisfaction compared to young patients [24]. Differences in sample size, physiotherapist’s skill, and facility might contribute to the satisfaction difference between the studies. Likewise, the overall Patient satisfaction in our study was lower compared to studies done in India(100% satisfied or completely satisfied) [12], Islamabad(91.2% satisfied or very satisfied) [6], and Saudi (70.9%) [20]. High-quality physiotherapy service with senior professionals in Saudi hospitals, the smaller sample size in India, and Islamabad, and different outcome measurement tools in Islamabad might be contributed to these visible differences.

On the contrary, our results showed a high satisfaction level compared to Pakistan (35.1%) [25]. The possible contributing factors for this difference may include a lack of similarity in study participants’ sociodemographic characteristics and clinical factors like professionalism. Most of the participants in Pakistan were treated by health workers who were not qualified professional physiotherapists like physiotherapy assistants and trainees and these may be the responsible factors for the inconsistency of results.

In the present study participants were most satisfied with the item ‘My therapist listens to my concern’’ and this finding was agreed with a study done in Kenya [23], [19] but this item was the least satisfying item in Srilanka [15]. Patients' overall satisfaction and their chance of returning back for future care were shown a very strong connection and this finding is because of the nature of patients’ tendency to recommend and return back for future service in the clinic where they were satisfied [26]. In our study participants gave more value to quality time and specifically to the Therapist’s ability to listen to their concerns. The reason behind this strong connection may be due to the chance of having a good relationship and building trust between patient and therapists is as high as the contact hour increase and ultimately this rise the likelihood of satisfaction among patients [27].

Regarding factors associated with overall satisfaction, pain level, longitudinal continuity of care, post-treatment health status, and referral system were significantly associated with overall satisfaction among physiotherapy outpatient attendees.

Study participants who were treated by the same (one) Physiotherapist throughout the course of treatment were 3.02 more satisfied compared to participants who were treated by Two and more physiotherapists. The significant association of Longitudinal continuity of care with overall satisfaction in our study was supported by a study done in Nepal [15], Canada [7], the USA [21], and the UK [28]. The possible reason for this association is explained by the fact that when patients are treated by one of the same therapists from the beginning the patent therapist relationship will develop and the patient’s chance of satisfaction will increase [21].

In our Study participants who rate their health status as better after starting treatment were 3.76 more satisfied compared to participants who claim their health status as worse or no change after treatment based on self-rating improvement as an outcome. This finding was supported by a study done in Canada [7], Spain [29], Saudi Arabia [20], and the USA [30]. The reason behind this relationship was explained by most of the patients who went to clinics or hospitals were to resolve from pain or problem they were suffering from and the main and the first priority they gave was the outcome or improvement [31].

Among study participants who complained of pain, those who labeled their pain level as low were 5.59 more satisfied compared to participants who had severe pain. This inverse relationship between pain level and patient satisfaction was supported by other studies [32–34]. The association between perceived pain level and dissatisfaction was explored by many findings. Most of the time Patients who complain of pain are exposed to stress and eventually to depression and this obscures patient satisfaction even after having excellent service from clinics or hospitals.

This study was one of the biggest of its kind in our country and it can serve as a cornerstone for responsible health policymakers to shift toward a client-centered approach which is the current widely accepted approach to increasing the efficacy of health services and patient adherence. Our study suggests patients give priority to pain reduction to secure patient satisfaction thus physiotherapists need to work on pain reduction, moreover, the present study witnessed therapists can ensure patient satisfaction by encouraging them to conduct the treatment session with the same therapist and targeting improving the patient health status in general. Finally, this study can be a baseline for future studies and health sectors can benefit from periodic satisfaction studies to increase the service quality.

## Conclusion

Based on our findings half of the participants were satisfied with outpatient physiotherapy service in Amhara region Comprehensive Specialized hospitals. Patients with improved health status compared to their prior health status and patient who had been treated with the same therapists throughout the session had a higher likelihood of satisfaction. Patients with less pain severity had a high probability of satisfaction and it is better if health providers better give infancies on the reduction of pain.

### Strength and limitation of the study

This is the first study in this study area in Ethiopia, in addition, the study used a multi-institutional cross-sectional study in order to encompass as many as a possible study participant. This makes the study conclusive. However, this study did not include a qualitative part of the study which is important in identifying specific factors affecting overall patient satisfaction.

## Data Availability

The manuscript contains all of the data that is important to our findings. Requests for more information about the dataset and questions about data sharing will be handled based on a fair request to yisakgr@gmail.com.

## Abbreviations

AOR: Adjusted Odd Ratio
CI: Confidence Interval
COR: Crude Odd Ratio
DCSH: Dessie Comprehensive Specialized Hospital
FHCSH: Felege Hiwot Comprehensive Specialized Hospital
OPD: Out Patient Department
OR: Odd Ratio
PT: Physiotherapist
SD: Standard Deviation
SPSS: Statistical Package for the Social Sciences
UK: United Kingdom
UOGCSH: University of Gondar Comprehensive Specialized Hospital
USA: United State of America
